# Obstetric Pharmacokinetic Dosing Studies are Urgently Needed

**DOI:** 10.3389/fped.2014.00009

**Published:** 2014-02-11

**Authors:** Shelley A. McCormack, Brookie M. Best

**Affiliations:** ^1^Pediatrics Department, School of Medicine, Rady Children’s Hospital San Diego, University of California San Diego, San Diego, CA, USA; ^2^Skaggs School of Pharmacy and Pharmaceutical Sciences, University of California San Diego, La Jolla, CA, USA

**Keywords:** pharmacology, pharmacokinetics, pregnancy, obstetrics, maternal–fetal

## Abstract

Use of pharmacotherapy during pregnancy is common and increasing. Physiologic changes during pregnancy may significantly alter the overall systemic drug exposure, necessitating dose changes. A search of PubMed for pharmacokinetic clinical trials showed 494 publications during pregnancy out of 35,921 total pharmacokinetic published studies (1.29%), from the late 1960s through August 31, 2013. Closer examination of pharmacokinetic studies in pregnant women published since 2008 (81 studies) revealed that about a third of the trials were for treatment of acute labor and delivery issues, a third included studies of infectious disease treatment during pregnancy, and the remaining third were for varied ante-partum indications. Approximately, two-thirds of these recent studies were primarily funded by government agencies worldwide, one-quarter were supported by private non-profit foundations or combinations of government and private funding, and slightly <10% were supported by pharmaceutical industry. As highlighted in this review, vast gaps exist in pharmacology information and evidence for appropriate dosing of medications in pregnant women. This lack of knowledge and understanding of drug disposition throughout pregnancy place both the mother and the fetus at risk for avoidable therapeutic misadventures – suboptimal efficacy or excess toxicity – with medication use in pregnancy. Increased efforts to perform and support obstetric dosing and pharmacokinetic studies are greatly needed.

## Introduction

Use of pharmacotherapy during pregnancy is common and increasing. From 1976 to 2008, the average number of medications (prescription and non-prescription) used during the first trimester increased from 1.6 to 2.6; this is an increase of approximately 60% (1). More recently, from 2006 to 2008 over 80% of women reported using at least one medication during the first trimester and over 90% reported using at least one medication at any point during the pregnancy (1). Given that during this time frame approximately 6.6 million pregnancies occurred per year in the United States (U.S.) (2), the issue of pharmacotherapy during pregnancy clearly has widespread relevance.

Increased use of medications during the first trimester is particularly concerning as this is a crucial period for organogenesis and, further, many women may be unaware of their pregnancy during this time. A recent review of 172 drugs approved by the Food and Drug Administration (FDA) from 2000 to 2010, conducted using the Teratogen Information System (TERIS), revealed insufficient data to determine teratogenic risk for nearly 98% of these drugs; over 70% actually had no pregnancy-related data available (3). Understanding teratogenic risk is important yet unfortunately understudied, especially for newer agents.

Despite the lack of safety data, often medication administration during pregnancy is unavoidable, with the benefits of treatment outweighing the possible risks. When medications need to be used during pregnancy, another concerning issue is the deficit of knowledge as to the proper dosing of pharmacotherapy during pregnancy. Understanding how to dose medications during pregnancy is crucial in order to ensure that the dose achieves therapeutic but not toxic plasma concentrations for both maternal and fetal wellbeing. Given the high and increasing rates of pharmacotherapy usage during pregnancy, gaps in pharmacology and appropriate dosing knowledge in pregnant women place both the mother and the fetus at risk for avoidable therapeutic misadventures.

The many physiologic changes throughout pregnancy affect pharmacokinetic parameters for pharmacotherapy administered during this time. The four primary processes of concern in pharmacokinetics, that is absorption, distribution, metabolism, and excretion, may all be influenced. Such pregnancy-related changes include changes in gastrointestinal motility and pH impacting absorption, expansion of total body water and plasma volume, and decreased concentrations of drug binding proteins affecting distribution, changes in drug metabolism rates by cytochrome P450 (CYP) and other metabolizing and transport enzymes, and increased glomerular filtration rate affecting urinary excretion (4). These various changes are not constant but rather fluctuate according to different patterns throughout the timeframe of pregnancy (4). These physiologic changes may have additive, synergistic, or competing effects on overall drug exposure. The clinical significance of these changes varies by drug, depending on the magnitude of the effect and the therapeutic window of the drug. The impact on overall drug exposure for any given drug is difficult to predict. To appropriately and safely dose drugs during pregnancy, these pharmacokinetic changes must be understood. Determination of the corresponding pharmacodynamic changes in pregnancy is also essential to understanding the pharmacokinetic changes in terms of implications for dosing. The objective of this focused review is to focus on the pharmacokinetic aspect to examine trends over time in the availability of maternal pharmacokinetic and dosing data for medications during pregnancy, to describe the drug classes or disease states, which have been studied for dosing determination to some degree during pregnancy, and to characterize sources of support for pharmacokinetic studies in pregnant women.

## Methods

A literature search was conducted within PubMed to assess the number of pharmacokinetic trials in pregnancy conducted over time and to determine which classes of drugs have been studied. A search of PubMed was performed for the terms “pharmacokinetics” and “pregnancy” with filters for “human” (species) and publication date through August 31, 2013 with no start date specified. “Pharmacokinetics” and “pregnancy” were not entered as MeSH terms after finding that many articles directly addressing pharmacokinetics were not indexed to have this as a MeSH term but rather had something more specific listed as a MeSH term instead, such as “adenine/pharmacokinetics.” A publication type filter was used for “clinical trial” and, separately, to search for all types except “case report” and “review.”

The results from this search strategy were stratified by year. Articles with electronic publication (e-publication) ahead of print were categorized in the year of print publication and not e-publication to avoid double-counting. All of the results categorized as clinical trials beginning with January 1, 2008 were more closely examined as follows. Those articles unrelated to the actual time period of pregnancy, despite being important to the larger field of women’s health, were excluded for the purposes of this review. Examples of such excluded topics include *in vitro* fertilization (IVF) treatments, breastfeeding and other postpartum issues, and sexually transmitted diseases outside of pregnancy. Articles pertaining to pregnancy but unrelated to pharmacotherapy, including environmental exposures, were also excluded. Finally, articles completely unrelated to pregnancy or humans and *in vitro* studies that appeared in the search results were excluded.

Additionally, a list of publications affiliated with the Obstetric-Fetal Pharmacology Research Unit (OPRU) network, provided on their website, was reviewed for additional publications and to test the completeness of the PubMed search strategy. Publications from 2008 through August 31, 2013 were manually reviewed for inclusion based on the same criteria as described above for the PubMed search results.

The non-excluded results from both the clinical trial PubMed and OPRU list searches were classified into broad categories as follows: (1) medication administration and parameter measurements occurring exclusively during labor and delivery and typically involving pain control or infectious disease (ID) prophylaxis [antiretrovirals (ARVs), antibiotics], (2) medications for treatment or prevention of pre-eclampsia or hemolysis, elevated liver enzymes, low platelets (HELLP) syndrome, and tocolytic therapy, (3) ARV regimens during pregnancy excluding one-time doses administered during labor, (4) non-HIV ID pharmacotherapy during pregnancy but again excluding doses given solely around the time of labor for prophylactic purposes, (5) vitamins and minerals during pregnancy (except magnesium for pre-eclampsia), and (6) studies of medications during pregnancy that do not fit into the other categories. Of the described six categories, categories 1–2 include studies focusing on acute and subacute conditions surrounding the time of delivery. Categories 3–6 focus on conditions requiring treatment at any time throughout pregnancy. Most studies involving a single dose at time of labor and delivery were placed in categories 1–2. However, a few such studies were placed into the categories 3–6 if they involved drug administration immediately prior to delivery but in a patient with scheduled cesarean and no onset of labor. This decision was made because the goal of such studies was in part to determine third-trimester (pre-labor) pharmacokinetic parameters. Since the dose was administered so close to delivery in such cases, fetal exposure could simultaneously be ascertained.

For the studies published after January 1, 2008, primary sources of funding were determined where possible. Acknowledgments and listings of funding support in the full texts of the articles were examined to categorize primary funding sources.

Finally, out of concern that the PubMed “clinical trial” filter may exclude some otherwise relevant articles, a filter for all publication types except “review” and “case report” was placed and a thorough manual review was performed for 2012 and 2013 (through August 31). This was performed to ascertain the rough proportion of total articles captured utilizing the “clinical trial” filter and allow for potential extrapolation.

## Results

**Figure 1:**
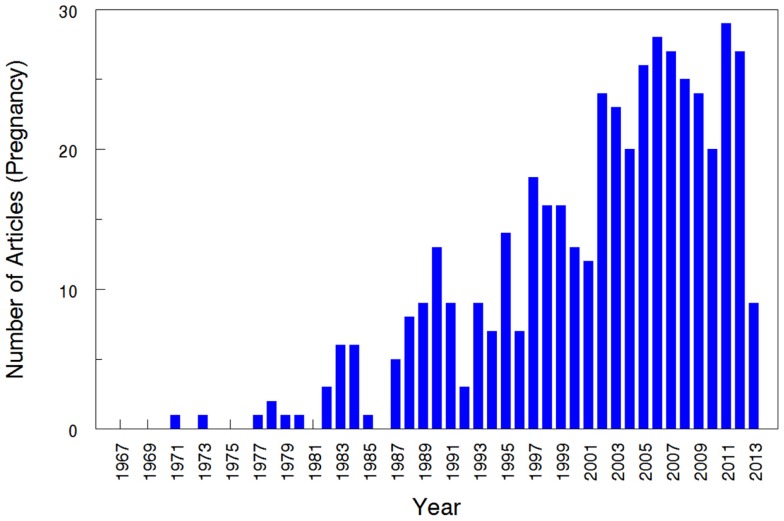
**Number of pharmacokinetic clinical trials conducted in pregnancy**. The year 2013 includes those articles index for MEDLINE by August 31, 2013 only. This figure displays the absolute number of articles meeting the search terms, displayed by year.

**Figure 2:**
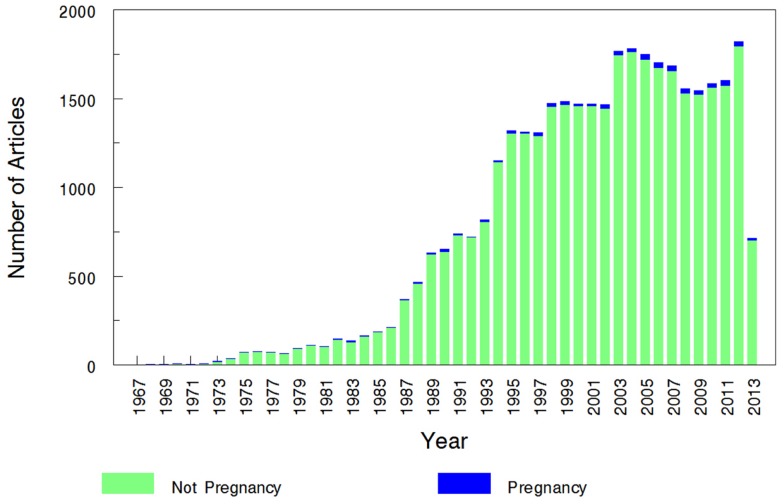
**Number of pregnancy and non-pregnancy pharmacokinetic clinical trials**. The year 2013 includes those articles index for MEDLINE by August 31, 2013 only. This figure displays the number of pregnancy pharmacokinetic articles along with all pharmacokinetic articles (pregnancy search term excluded) found to otherwise match the search terms specified.

**Figure 3:**
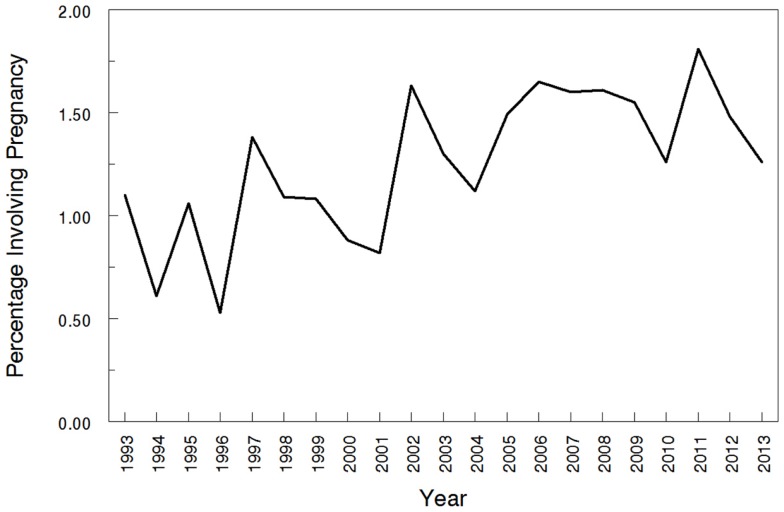
**Proportions of pharmacokinetic clinical trials conducted in pregnancy**. The year 2013 includes those articles index for MEDLINE by August 31, 2013 only. This figure shows the percentage of pregnancy pharmacokinetic articles compared to all pharmacokinetic articles published since the early 1990s.

The overall search of PubMed as described and using the “clinical trial” filter yielded 464 results. Without the term “pregnancy” this same search had 35,921 results, indicating that only 1.29% (464/35,921) of the pharmacokinetic studies found in the search involved pregnant women. As can be visualized in Figures 1 and 2, absolute number of pharmacokinetic studies, both with and without pregnancy data, have steadily increased over the past four decades with particularly large increases from the mid-1980s to mid-1990s. However, as shown in Figure 3 the proportion of these studies involving pregnancy has been relatively constant since around 1990; years prior to the mid-1980s had significant fluctuations in this proportion as expected given the low absolute values. The year 2013 is displayed in Figure 1 for informational purposes; note that it only includes those articles indexed in MEDLINE by August 31, 2013. The number of articles for 2013 were not adjusted to account for the partial year as scaling for 8 of 12 months did not seem reasonable given the inconsistent multi-month delay between publication and appearance in PubMed. When searching for all except “review” and “case report” publication types, the total number of articles was 156,539 with 3633 of these in pregnancy (2.3%). However, as per the manual review of a subset of this broader search described below, most of the articles when searching by “clinical trial” were relevant whereas most found by the broader search were excluded. Hence, the percentage of 1.29 from the narrower search is likely more reliable and the findings from the broader search were not further stratified by year. Additional pregnancy studies identified through the OPRU listing search are not included in these overall percentages as there was not a comparable method available to check for additional studies in PubMed associated with the overall (pregnancy plus non-pregnancy) count.

When narrowing the above search, limited to “clinical trial,” to the time frame of January 1, 2008 to August 31, 2013, 134 articles remained. Of these, 59 were excluded based on the criteria specified in the Section “Methods” above, leaving 75 for further classification. Of the 75 remaining studies, 8 were drug studies that did not measure drug concentrations and appeared unrelated to pharmacokinetics despite this being one of the search terms. These studies typically involved measurement of an outcome (e.g., change in blood pressure) in response to drug dosing adjustments without measuring the drug concentration itself. Given that these studies did not meet the exclusion criteria and still contribute to improved pharmacotherapy in pregnancy, they were included in this review but noted accordingly in Table 1.

**Table 1:** **Classification of search results for recent clinical trials of pharmacokinetics and pregnancy**.

Year	Total (PubMed + OPRU)	Acute/subacute		Chronic			
		Intra-partum	Late ante-partum	ARVs ante-partum	Other ID ante-partum	Vitamins/minerals ante-partum	Other medications ante-partum
2013 (1/1–8/31 only)	9	3	2	2	0	1	1
2012	14	2	2^a^	4	1	1^a^	4
2011	17	4	0	4	4	0	5
2010	11	4^b^	1	1	2	0	3
2009	16	3	1	2	2	1	7^b^
2008	14	3^a^	1	3	2	0	5^a^
Total (1/2008–8/2013)	81	19	7	16	11	3	25

*Data for 2013 only includes those articles indexed for MEDLINE as “clinical trial” (publication type) by August 31, 2013. The “intra-partum” category includes medications given at the time of delivery, typically for pain control or infectious disease prophylaxis. The “late ante-partum” category includes pharmacotherapy given for pre-eclampsia; management of syndrome of hemolysis, elevated liver enzymes, and low platelets (HELLP); or to induce tocolysis. The remaining categories include medications administered earlier in pregnancy (i.e., prior to labor/delivery) only*.

*ARVs, antiretrovirals; ID, infectious disease. References for each column: intra-partum (5–23), late ante-partum (24–30), ARVs (31–46), other ID (47–57), vitamins/minerals (58–60), other medications (61–85)*.

*^a^One study in this category did not measure drug concentrations*.

*^b^Two studies in this category did not measure drug concentrations*.

Six additional relevant studies, approximately one per year, were identified by review of the OPRU studies listing. These were not found by PubMed only because they were not indexed as “clinical trial” (publication type), but upon careful review “clinical trial” indexing would have been appropriate. This resulted in a total of 81 studies included from the two search methods.

**Figure 4:**
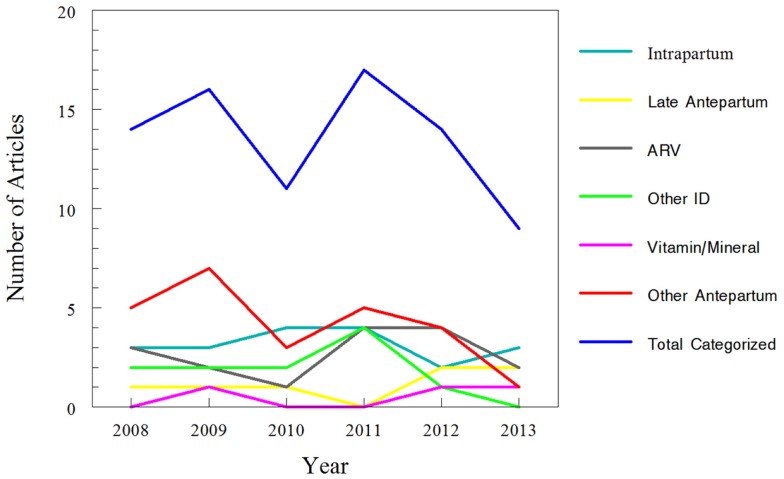
**Trends for classification of search results for recent clinical trials of pharmacokinetics and pregnancy**. Data for 2013 only includes those articles indexed for MEDLINE by August 31, 2013. The “intra-partum” category includes medications given at the time of delivery, typically for pain control or infectious disease prophylaxis. The “late ante-partum” category includes pharmacotherapy given for pre-eclampsia; management of syndrome of hemolysis, elevated liver enzymes, and low platelets (HELLP); or to induce tocolysis. The remaining categories include medications administered earlier in pregnancy (i.e., prior to labor/delivery) only.

Approximately one-third of studies (26/81) classified were focused on pharmacokinetic issues during or immediately preceding labor; specifically, 19 studies were during labor and delivery and 7 more focused on pre-eclampsia and HELLP management prior to delivery or tocolytics to delay delivery. In comparison, 55 studies addressed medications given chronically during pregnancy. Of the chronic medications, 16 of the 55 studies were for HIV treatment and vertical transmission prevention. More detailed results of this classification are displayed in Table 1 and Figure 4.

Within the non-HIV ID category, the sub-classification by year is as follows. No articles have been published yet in 2013. In 2012 azithromycin was studied (47). 2011 included one study of metronidazole for bacterial vaginosis (48), one study of oseltamivir (49), and two studies of anti-malarial regimens (50, 51). In 2010 and 2009 two studies of anti-malarial regimens in pregnancy were published each year (52–55). 2008 had one study of an anti-malarial regimen (56) and one of amoxicillin in preterm premature rupture of membranes (57).

The final category, containing all drug classes given throughout pregnancy but not related to IDs, contained a sub-classification of available studies by year as follows. In the first 8 months of 2013, the only medication studied was doxylamine/pyridoxine for nausea/vomiting in pregnancy (61). In 2012, these medications included metformin for Type 2 diabetes in obese pregnant women (62), tacrolimus (63), 17-hydroxyprogesterone for prevention of preterm labor in patients at risk (64), and doxorubicin and ifosfamide for high-grade sarcomas during pregnancy (65). In 2011, the medications were buprenorphine for opioid dependence treatment (66), two studies of methadone written by the same authors (67, 68), intravenous immunoglobulin (IVIG) (69), and labetalol (70). 2010 had one study of fluoxetine (71), one of metformin (72), and one of glucose kinetics (73). In 2009, studies addressed dydrogesterone to prevent miscarriage in patients with second trimester vaginal bleeding (74), doxylamine/pyridoxine for nausea/vomiting of pregnancy (75), sumatriptan/naratriptan (76), safety of serotonin reuptake inhibitors (SSRIs)/serotonin norepinephrine reuptake inhibitors (SNRIs) (77), glyburide for gestational diabetes (78), lamotrigine for epilepsy (79), and clonidine (80). The drugs studied in 2008 were sertraline (81), enoxaparin (82), glyburide (83), aspirin in patients whose pregnancy was achieved via IVF (84), and finally midazolam and digoxin (as probe substrates studying CYP3A enzymes and P-glycoprotein) (85).

When broadening the search criteria to include all publication types except for case reports and reviews, 634 additional articles were found from January 1, 2008 through August 31, 2013. Narrowing from January 1, 2012 to August 31, 2013 resulted in 180 additional articles. When manually reviewing each of these 180 results for original data relevant to dosing during pregnancy, only 18 were relevant (14 in 2012 and 4 through August 2013). Combining with the 23 relevant articles found by searching for “clinical trial” yields a total of 41 relevant articles during the time period and indicates that the broader count for the other years reported in Table 1 may be increased by approximately 44%. The additional relevant studies included three studies related to intra-partum pain management (86–88), four on ARVs ante-partum (89–92), six on antimalarials (93–98) of which many are by the same authors, one on vitamin D (99), and five on other ante-partum pharmacotherapy. Of these latter four studies, one study addressed anti-D IgG dosing (100), one addressed anti-tumor necrosis factor for inflammatory bowel disease (101), and two modeled activities of CYP enzymes (102, 103).

**Figure 5:**
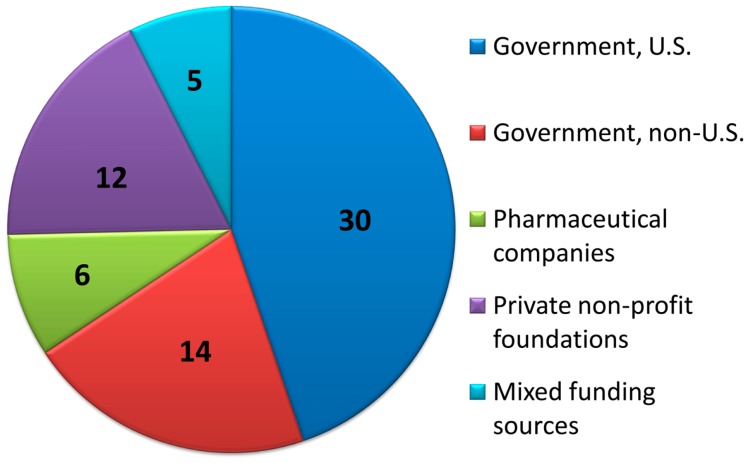
**Distribution of primary funding sources**. For clinical trials with funding source information provided, the number of studies primarily funded by each source is displayed.

Primary funding sources for the clinical trials published since January 1, 2008 were determined if available. Of the 81 articles included, all except for 13 articles stated their source of funding. See Figure 5 for the approximate distribution of funding sources.

Of the 68 clinical trials with explicit funding information, 30/68 (44%) were funded via U.S. government organizations; one article was funded by U.S. Department of Agriculture (USDA) and 29 were by various institutes of the National Institutes of Health (NIH). The primary NIH institutes involved were the National Institute of Allergy and Infectious Diseases (NIAID) and the *Eunice Kennedy Shriver* National Institute of Child Health and Human Development (NICHD); other supporting institutes included the National Institute of Diabetes and Digestive and Kidney Diseases (NIDDK), the National Institute of Mental Health (NIMH), the National Institute on Drug Abuse (NIDA), and the National Center for Research Resources (NCRR). The OPRU network, funded by the NICHD was, as per their listing of affiliated publications, associated with 11 of the studies (26, 27, 48, 49, 61, 63, 64, 72, 78, 80, 83). Some of these were explicitly sponsored by OPRU as per the author listing or funding acknowledgment; others stated a non-governmental source of funding but presumably were included in this listing based on author affiliation.

Another 14/68 (21%) of the studies were funded by governmental funding from outside the U.S., including Brazil (five studies), Australia (two studies), Belgium, Canada, Germany, Hong Kong, Japan, Netherlands, and Switzerland. An additional study was a joint effort of government funding from the U.S. (NIH), France, and Thailand.

Drug companies directly funded 6/68 (8.8%) of studies, excluding legally independent foundations that receive support from drug companies. Private non-profit foundations (U.S. and abroad) funded 12/68 (18%) of the studies, and combinations of public and private funding were responsible for the remaining 5/68 (7%) studies.

## Discussion

This review found that only 1.29% of pharmacokinetic clinical trials indexed in PubMed provide data for pregnant women. A search for pregnancy rates within the National Vital Statistics Reports, at present containing data through 2008, revealed a yearly pregnancy rate for females in the U.S., ranging from 10.4 to 10.7% (2). This would roughly correspond to a population of yearly pregnancy rate ranging from 5.2 to 5.4%. These rates are up to five times higher than the rates of pregnancy-related studies available during this same time frame, which were 1.1–1.6% as per this review. The decision to use medications during pregnancy should be based on a complete understanding of the risks and benefits and should include an understanding of how to optimally dose these medications in this population. Unfortunately, the knowledge of the risks, benefits, and optimal dosing are often incomplete; during pregnancy, these knowledge gaps in many cases are vast.

This review found that relative to other drug classes, the ARVs have a larger number (16/81:20% since 2008) of pharmacokinetic clinical trials during pregnancy. This is perhaps in part due to explicit devotion of resources and the medical community’s agreement and public acceptability that these medications are not only for maternal wellbeing but to prevent transmission of HIV to the fetus. These HIV studies have found that doses for some medications but not others do need to be altered during pregnancy. The findings of the pharmacology studies in pregnant women with HIV highlight the need for studies of the multitudes of other medications and drug classes being used by pregnant women.

Over time many women are postponing childbearing to an older age (2), increasing the risk of concomitant chronic health issues throughout pregnancy. Further, advances in reproductive technology can potentially enable women to become pregnant who would not have been healthy enough otherwise. Certain chronic conditions such as Type 2 diabetes are frequently appearing at younger ages than they have in the past and may exist prior to pregnancy (104–106). All of these issues increase the risk that a pregnant female will have a chronic medical condition prior to pregnancy that necessitates chronic pharmacotherapy during pregnancy. Such pharmacotherapy requires knowledge of the proper dosing in order to achieve the proper drug concentrations for efficacious management of the condition and to prevent poor fetal outcomes associated with poor maternal disease control.

This raises the question of which medical conditions are seen most frequently in the childbearing population and if sufficient evidence exists to treat these conditions appropriately during pregnancy. A recent study surveyed mothers to determine which medications they had used during the first trimester of pregnancy (107). The majority of oral medications in the list generated by that study fell into the categories of antibiotics, analgesics, anti-emetics, diabetes treatment (metformin and insulin), and psychiatric medications (primarily antidepressants) (107). Of these, data were significantly lacking for the psychiatric medications as well as for some of the antibiotics. Proper treatment of both infections and psychiatric illnesses is crucial in pregnancy for both maternal and fetal wellbeing – perhaps even more crucial than in non-pregnant populations.

For the specific psychiatric example of depression, prevalence of depression from 2007 to 2010 in females ages 18–39 was approximately 10% (108); this encompasses most of the childbearing years. In the next age category, ages 40–59, this rate was similar at 12% (108). However, for ages ≥60, the depression rate in females drops to 7% (108). Based on these prevalence rates, the common usage of antidepressants during pregnancy is not surprising. However, over the time period closely examined in this review (January 2008 to August 2013), only three pharmacokinetic studies were published pertaining to psychiatric medications. These studies only addressed selective SSRIs and one SNRI. Further, while all three studies measured maternal SSRI or SNRI concentrations, only two (71, 81) were designed to determine maternal pharmacokinetic parameters and proper dosing. The third focused on correlating the maternal drug concentrations to cord concentrations and neonatal behavioral outcomes (77). Increased inclusion of the pregnant population in pharmacokinetic studies of antidepressants is a necessary change in order to address this knowledge gap.

In addition to the need for increased awareness of the scope of this problem of insufficient pharmacokinetic trials during pregnancy, increased incentive for government and non-government funding of these trials is also needed. Currently, the pharmaceutical industry has no incentive or mandate to fund trials that include pregnant patients as part of the drug approval process, and, as revealed, the drug companies were the primary sponsors of only 8.8% of the trials discussed. One possible solution to this issue would be to pass legislation providing the FDA with the authority to mandate pharmaceutical companies to complete testing of any medications that would likely be used by the pregnant population. An additional approach would be increasing the support of pharmacokinetic trials in pregnancy by private and by government-funded organizations. Government support for existing disease-specific or pharmacology-related clinical research networks charged to perform obstetric-fetal pharmacology studies could be increased to allow for more sites, more basic/translational studies, and more clinical trials. Another approach could be that each NIH institute (NIDDK, NHLBI, NIAID, NICHD, NIMH, etc.) maintain a certain percentage of pregnancy dosing-related studies within its portfolio of supported studies.

In moving forward to incentivize, mandate, or simply encourage and support an increased number of pharmacokinetic studies during pregnancy, the field of pediatrics provides one potential model. Until recent years, similarly, dosing information was lacking for most medications frequently administered to pediatric patients. This problem is now slowly improving through a series of government-based incentives and mandates (109–111). Both the pediatric and pregnant populations face a reluctance to perform clinical trials, in part due to legitimate ethical concerns. However, as pharmacotherapy must often be given in either group, the result of *not* testing in these groups appears to be gradually gaining recognition as an equally legitimate ethical concern. Hopefully, the field of obstetrical pharmacology will be able to make similar or more progress as the pediatric pharmacology field in the near future.

This brief, focused review did not capture all pharmacokinetic studies during the time frame listed, as evidenced by the 44% increase in studies when the formal “clinical trial” filter was removed. However, this was not designed as a comprehensive meta-analysis. Rather, the goal was to provide a general overview of trends over time and for various classes of drugs in order to raise awareness of gaps in the literature and focus future efforts accordingly. The inevitable shortcomings of the search strategy do not change the overall findings relative to this goal. This review found that the number of pharmacokinetic studies performed in pregnant women are exceedingly low, despite the fact that medication use during pregnancy is widespread and increasing.

This review does not focus on pharmacodynamic studies. A brief search using this term yields 828 studies in pregnancy since 2012 even with the restrictive “clinical trial” filter, but many of the automated findings are surprisingly irrelevant to pharmacotherapy and very few are related to dose determination. Many of the dosing studies are for treatment given only at the time of delivery, such as pain control, for which outcome can be promptly assessed. It is likely that there is a shortage of pharmacodynamic studies in pregnancy, but further investigation is needed to ascertain the scope of this problem. This issue is crucial, as without accompanying pharmacodynamic data the pharmacokinetic studies’ usefulness is limited.

An understanding of the clinical pharmacology of medications during pregnancy is critical to develop optimal dosing regimens. Inappropriate dosing of medications during pregnancy can have minor to profound adverse consequences for both the mother and the fetus. Increased number of studies and sources of support for pharmacokinetic studies in pregnant women are greatly needed.

## Conflict of Interest Statement

The authors declare that the research was conducted in the absence of any commercial or financial relationships that could be construed as a potential conflict of interest.
